# Permeation and Rectification in Canonical Transient Receptor Potential-6 (TRPC6) Channels

**DOI:** 10.3389/fphys.2018.01055

**Published:** 2018-08-03

**Authors:** Stuart E. Dryer, Eun Young Kim

**Affiliations:** ^1^Department of Biology and Biochemistry, University of Houston, Houston, TX, United States; ^2^Department of Medicine, Division of Nephrology, Baylor College of Medicine, Houston, TX, United States

**Keywords:** TRPC6, calcium signaling, podocytes, mesangial cells, vascular smooth muscle, TRP channels, G protein signaling, mechanotransduction

## Abstract

Transient receptor potential-6 channels are widely expressed cation channels that play a role in regulating Ca^2+^ dynamics, especially during G protein-coupled receptor signaling. The permeation of cations through TRPC6 is complex and the relative permeability to Ca^2+^ relative to monovalent cations appears to be highly voltage-dependent and is reduced upon membrane depolarization. Many investigators have observed complex current-voltage (I-V) relationships in recordings of TRPC6 channels, which often manifest as flattening of I-V curves between 0 and +40 mV and negative to -60 mV. These features are especially common in recordings from TRPC6 channels expressed in heterologous expression systems. Indeed, it is sometimes argued that marked rectification at both negative and positive membrane potentials is a defining feature of TRPC6, and that recordings in which these features are reduced or absent cannot reflect activity of TRPC6. Here we present a review of the literature to show that complex rectification is not seen in every cell type expressing TRPC6, even when comparing recordings made from the same groups of investigators, or in recordings from what is nominally the same heterologous expression system. Therefore other criteria, such as gene knockout or knockdown, or the use of newly emerging selective blockers, must be used to ascertain that a given current reflects activity of endogenously expressed TRPC6 channels. We also discuss the possibility that complex rectification may not be an intrinsic property of TRPC6 in cells where it is observed, and may instead reflect presence of endogenous substances that cause voltage-dependent inhibition of the channels.

## General Features of Trpc6 Channels and Their Modes of Activation

Canonical transient receptor potential-6 (TRPC6) channels are cation channels expressed in most mammalian organs and in a host of cell types, including smooth muscle, epithelia, and immune cells, and other cell types, including several important renal cell types (Bouron et al., 2016). They are of particular interest to renal physiologists and nephrologists owing to their role in driving renal pathology (Winn et al., 2005; Kim et al., 2018) and in modulation of mesangial cells (Ma et al., 2016) and regulation of vascular smooth muscle tone (Gonzalez-Cobos and Trebak, 2010). TRPC6 channels are most notable for their participation in G-protein-coupled receptor (GPCR) signaling pathways that contribute to Ca^2+^ dynamics in cells, although they can clearly participate in other pathways. TRPC6 is a member of a subfamily of TRP channels called “canonical" or “classical" because they bear the highest homology to the *Drosophila* TRP and TRPL channels that were discovered initially (Abramowitz et al., 2007). Within the TRPC family, TRPC6 is most similar to TRPC3 and TRPC7, and indeed can form functional heterotetramers with TRPC3 but does not generally form heteromers with TRPC4 or TRPC5 or more distantly related TRP channels in heterologous expression systems. In this regard, TRPC3 differs from TRPC6 in having higher levels of basal activation that has been shown to be a consequence of its dual glycosylation (Dietrich et al., 2003).

Transient receptor potential-6 subunits contain several distinct motifs in portions of the protein that extend into the cytosol. These include a series of ankyrin repeats in the cytosolic portion of the amino terminus (the so-called ankyrin-repeat domain) and a coiled-coil domain located closer to the first transmembrane domain. Those cytosolic motifs are thought to participate in protein interactions and are essential for tetrameric channel assembly (Lepage et al., 2006). In the cytosolic carboxy-terminus a so-called TRP domain is found in all TRPC6 channels, followed by a proline-rich motif, a putative calmodulin- and IP_3_ receptor-binding domain, and another coiled-coil domain. Those portions of the subunits are also thought to contribute to assembly of functional channels and probably mediate interactions with a number of other proteins (Bouron et al., 2016), for example podocin (Anderson et al., 2013). There are also six membrane-spanning α-helices, which includes the pore-forming domain, as with many other cationic channels.

The factors that cause activation of TRPC6 and other TRPC channels are complex, not well understood, and are in many cases controversial. The most extensively studied activation mode for TRPC6 entails diacylglycerol (DAG) detached from plasma membrane inositol phospholipids by phospholipase C as a result of GPCR signaling. Importantly, DAG analogs are able to activate TRPC6 even in excised inside-out membrane patches, suggesting that all components needed for TRPC6 activation are membrane-delimited (Hofmann et al., 1999). This further implies that DAG and its analogs cause activation in part by binding at or near TRPC6 channels already located in the plasma membrane. In this regard, some compounds that activate TRPC6, such as hyperforin and other acylphloroglucinol derivatives are rigid analogs of DAG (Leuner et al., 2007, 2010). Other endogenous lipids, such as 20-HETE can also increase activation of TRPC6 (Basora et al., 2003; Inoue et al., 2009; Roshanravan et al., 2016). 20-HETE is produced inside cells in pathways that include phospholipase A2 activation, but also occurs in extracellular space and in the circulation. In addition, the activity of TRPC6 is redox-sensitive, and reactive oxygen species (ROS) appear to increase the probability of opening of TRPC6 channels already in the plasma membrane. However, ROS also markedly increase the steady-state surface expression of TRPC6 (Graham et al., 2010; Kim et al., 2012). In this regard, GPCR signaling can cause a rapid insertion of TRPC6 into the plasma membrane (Cayouette et al., 2004), and ROS-dependent TRPC6 activation has been demonstrated for endogenous signaling systems (Kim et al., 2012, 2013; Anderson et al., 2014; Roshanravan and Dryer, 2014; Roshanravan et al., 2016). There is also evidence that TRPC6 can become active in response to mechanical deformation of the plasma membrane (Welsh et al., 2002; Dyachenko et al., 2009; Quick et al., 2012; Anderson et al., 2013; Seo et al., 2014; Nikolaev et al., 2016; Yamaguchi et al., 2017), although the mechanisms whereby this occurs are not well understood and probably depend on what cell type is considered (Inoue et al., 2009; Sharif-Naeini et al., 2010; Quick et al., 2012; Anderson et al., 2013; Wilson and Dryer, 2014). TRPC6 mobilization also occurs in response to activation of tyrosine kinase receptors, such those activated by insulin (Kim et al., 2012) and also in response to integrin activation (Kim et al., 2017).

## Cation Permeation Through Trpc6 Channels

The most salient feature of TRPC6 in terms of permeation is that it is a non-selective cationic channel. In landmark studies of cloned TRPC6 channels, Hofmann et al. (1999) observed that TRPC6 is permeable to Ca^2+^, Cs^+^, Na^+^, and K^+^. By measuring reversal potentials under various bi-ionic conditions in whole cell recordings, these workers found that TRPC6 channels were approximately five-fold more permeable to Ca^2+^ than to Na^+^ at voltages close to the reversal potentials used in their conditions (typically close to -10 mV). Consistent with this conclusion, these workers found that TRPC6 could mediate Ca^2+^ influx into a non-excitable heterologous expression system as measured using Fura-2. Complexities in Ca^2+^ permeation through TRPC6 were suggested by another early study by Inoue et al. (2001), as they observed markedly reduced outward current through TRPC6 (at positive membrane potentials) when Ca^2+^ was the primary cation in external solution compared to when Na^+^ was the main cation. This group subsequently showed that modest elevations of external Ca^2+^ could reduce inward current through TRPC6 when Na^+^ was also present, which suggested that at least part of the complex actions of Ca^2+^ on TRPC6 entail some degree of pore-blockade (while other effects to enhance TRPC6 function appeared to be mediated by Ca^2+^-calmodulin) (Shi et al., 2004).

A subsequent study examined TRPC6 blockade by Ca^2+^ in more detail by measuring macroscopic currents with perforated-patch methods while Ca^2+^ influx was simultaneously monitored with Fura-2 (Estacion et al., 2006). These workers obtained experimental data on relative Na^+^ and Ca^2+^ permeability consistent with barrier-and-well models with a single cation binding site in the channel pore located about 85% of the distance through the transmembrane electric field, separated by two relatively high energy barriers to permeation located close to the inner and outer limits of the pore. There are several physiological implications for the biophysical data and models that have been generated. Specifically, Ca^2+^ can permeate TRPC6 more readily at more negative membrane potentials, but membrane depolarization progressively causes TRPC6 to behave as a monovalent cation channel that is blocked by external Ca^2+^. If the primary role of TRPC6 is simply to allow receptor-mediated depolarization of a cell to the point where voltage-activated Ca^2+^ channels can drive the bulk of Ca^2+^ influx, whether or not TRPC6 is Ca^2+^-permeable probably doesn’t matter. This could be the situation in vascular smooth muscle, for example. However, in non-excitable cells, such as epithelia, where channels containing TRPC6 are suspected to drive the bulk of Ca^2+^ influx, then there should be a mechanism in place to limit the cell depolarization that occurs as a result of TRPC6 activation (for example voltage- and/or Ca^2+^-activated K^+^ channels should also be present). Alternatively, there may be some other process that enhances Ca^2+^ permeation and reduces pore block (possibly heteromerization with other TRPC subunits or other auxiliary subunits that would result in a slightly different pore structure). We have already noted that TRPC6 can form heteromeric channels with TRPC3, and the permeation properties of these heteromers are not known. In addition, we have observed that TRPC6 channels endogenously expressed in immortalized podocytes can immunoprecipitate with KCa1.1 channels (Kim et al., 2009). Since podocytes are non-excitable cells, this could provide a mechanism to limit depolarization evoked by TRPC6 activation. Thus, a complete understanding of the role of TRPC6 in the Ca^2+^ dynamics of any non-excitable cell will require understanding of the mechanisms that allow maintenance of Ca^2+^ permeation in physiological conditions, where membrane potential is free to fluctuate (in contrast to the voltage-clamped conditions widely used in experiments). TRPC6 also forms heteromers with TRPC1, which results in reduced Ca^2+^ permeability (Storch et al., 2012).

## Complex and Simple Rectification of Current Flowing Through Trpc6 Channels

**FIGURE 1:**
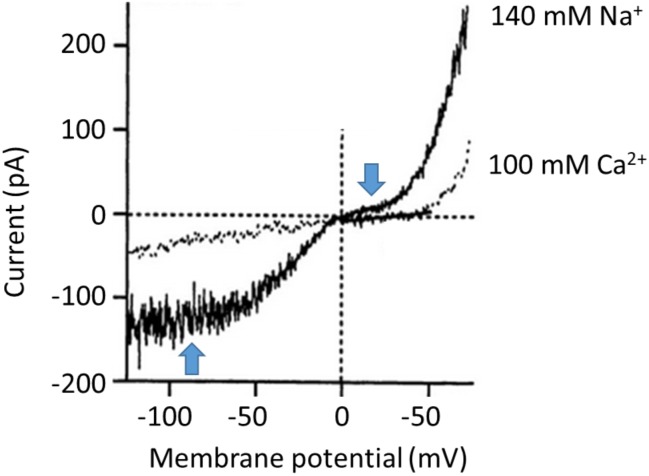
One of the best examples of the complex “double rectification” in the I-V curve of a TRPC6 channel originally published by Inoue et al. (2001). These are traces from a conventional whole-cell recording from TRPC6 channels heterologously expressed in HEK293 cells. The trace in gray shows recordings made when the external bath solution contained 100 mM Ca^2+^, whereas the trace in black shows recordings from the same cell made in a somewhat more physiological condition in which the bath solution contained 140 mM Na^+^ and no divalent cations were present. Note that currents in either direction are reduced when recordings are made with Ca^2+^ in the external solution, suggesting that it can partially inhibit the channels. Note also the complex shape of the I-V curve in the recording made with external Na^+^, which the authors of the paper described as “S-shaped.” We have added blue arrows to call attention to regions where there is flattening of the I-V curve, negative to –60 mV and between 0 and +40 mV. We have re-lettered the axes for clarity. Figure is used with permission from the publisher.

**FIGURE 2:**
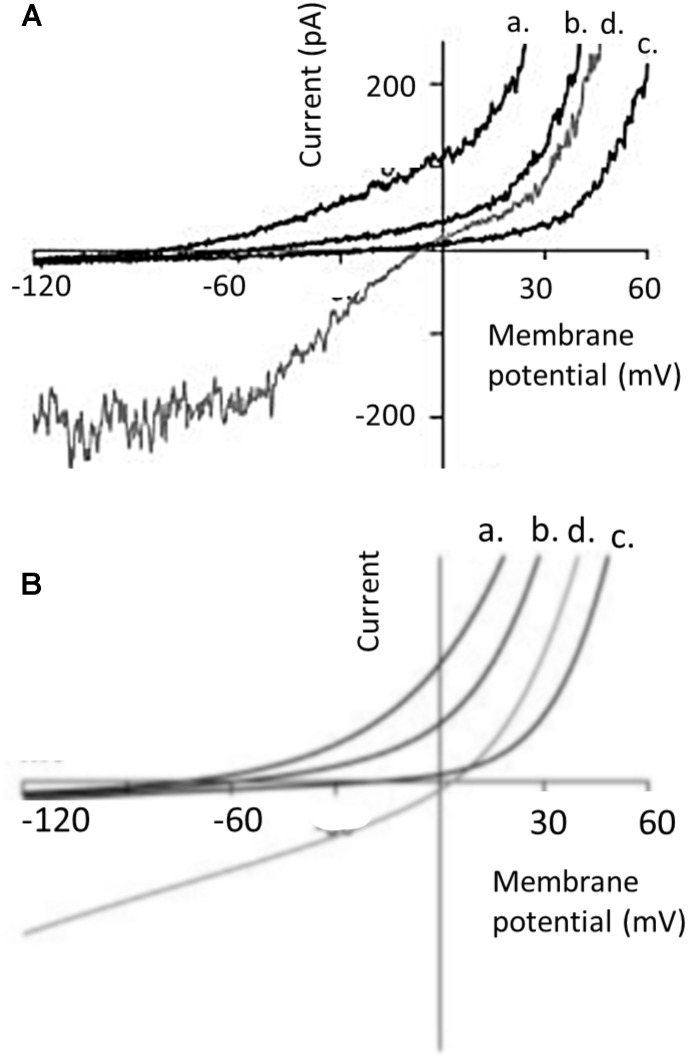
I-V curves in different external bath solutions from perforated patch whole-cell recording from TRPC6 channels heterologously expressed in HEK293 cells, and comparison to a barrier-and-well model, both originally published by Estacion et al. (2006). **(A)** Shows currents evoked by 100 μM OAG. Currents are shown in presence of a normal external solution containing 160 mM Na^+^, 5 mM K^+^, 2 mM Ca^2+^, and 1 mM Mg^2+^ (trace d, shown in gray); and in external solutions in which Na^+^ was replaced with *N*-methyl-*D*-glucamine and containing 0 mm Ca^2+^ (trace a), 2 mM Ca^2+^ (trace b), and 20 mM Ca^2+^ (trace c). Note the flattening of the I-V curve in normal external solutions (trace d) between –60 and –120 mV and also between 0 and +30 mV. Note also that Ca^2+^ in the bath solution produces concentration-dependent inhibition of outward currents. **(B)** I-V curve predicted for similar conditions from a barrier-and-well model with barriers at the entrances to the pores and energy well in the pores that binds Ca^2+^ more tightly than Na^+^. The model captures the inhibitory effect of Ca^2+^ on currents in either direction but does not capture flattening in the I-V curves. We have re-lettered the axes for clarity. Figure is used with permission from the publisher.

Since the initial studies of Hodgkin and Huxley on the mechanisms underlying excitability in the squid axon (Hodgkin and Huxley, 1952) it has been common for electrophysiologists to examine the amplitude of a current (*I*_M_) over a range of different membrane potentials (*V*_M_). The *V*_M_ can be varied by application of a series of pulses, or as a single ramp command in which *V*_M_ is varied relatively slowly at a constant d*V*_M_/dt. The later method is a convenient experimental design and yields good results as long as the ramp is not too fast (so that currents associated with the membrane capacitance are small and constant). The range of command potentials that can be used experimentally varies from cell to cell since not all cells can tolerate the same extremes of transmembrane electric fields without the membrane essentially melting. If the current *I*_M_ is plotted on the ordinate against *V*_M_ on the abscissa, the result is a so-called I-V curve. The shape of the I-V curve depends on many factors, including the concentrations of permeant ions on either side of the membrane, whether or not the channel exhibits voltage-dependent gating, the presence of possible charged substances that could block the pore, and the pathway that permeant ions must take en route through the pore itself. In real world experiments, it also depends on whether or not observed current reflects isolated activity of a homogenous population of channels. The simplest description of ion permeation through channels is the so-called constant field model (an idealized formulation akin to the natural gas law in the simplicity of its assumptions). This model ignores gating phenomena, and assumes a channel in which permeant ions are in contact with water at all times as they undergo free electrodiffusion, and do not interact with each other or in any significant way with the pore during permeation (Hille, 2001). In other words, the electric field that influences ions as they traverse the membrane is constant at all regions within the pore, and the permeation pathway is uniform. However since transmembrane current requires both a permeation pathway and mobile particles to carry charge, even constant field assumptions will yield curved I-V plots if the solutions on either side of the membrane are not symmetrical with respect to concentrations of permeant ions. Nearly all channels exhibit varying degrees of deviation from constant-field behavior, and striking examples of this have been reported for TRPC6. However, in the case of TRPC6 the nature of these deviations varies enormously from one report to another, even in experiments that appear to be done under similar conditions. In the rest of this commentary we will argue that the shape of an I-V curve cannot be used to ascertain whether or not a current is being mediated by channels containing essential TRPC6 subunits. The earliest studies on TRPC6 channels noted marked degrees of deviation from constant-field behavior. A widely cited example is the TRPC6 I-V curve reported by Inoue et al. (2001) based on recordings of cloned TRPC6 channels expressed heterologously in HEK293 cells (**Figure 1**). Heterologous expression of channels in HEK293 cells is a somewhat artificial system but one that is widely used because those cells are very easy to record from, allow very wide voltage ranges to be applied, and are well suited to automation for higher throughput analyses. In most cases TRPC6 channels are activated by stimulation of a GPCR that is also expressed heterologously (muscarinic receptors and histamine receptors have been widely used), although in some cased workers will use membrane permeable non-physiological DAGs, such as 1-oleoyl-2-acetyl-*sn*-glycerol (OAG) or 1-stearoyl-2-arachidonoyl-*sn*-glycerol (SAG) to activate the channels. It is often assumed that HEK293 cells don’t express confounding channels endogenously, although this may be a bad assumption for TRPC subfamily channels (Garcia and Schilling, 1997). There is also a somewhat uncritical implicit acceptance of the idea that cells that are called HEK293 in one laboratory are nearly identical to other cells with the same name that have been maintained for long periods of time in other laboratories, especially in cases where laboratories are using cells stably transfected with TRPC6. The I-V curves obtained by Inoue et al. (2001) in the presence of 140 mM external Na^+^ exhibit numerous changes in slope, sometimes referred to as “dual rectification.” We have added blue arrows to **Figure 1** to call attention to these features. There is a somewhat flat region at membrane potentials negative to -60 mV, an inflection around the reversal potential at 0 mV, a second flat region between 0 and +40 mV, and monotonic outward rectification at potentials positive to +40 mV. As noted earlier, Ca^2+^ does not carry current as effectively as Na^+^, and in the presence of Ca^2+^ outward current is reduced and the I-V curve is simpler. Hereafter we will focus on I-V curves obtained in external Na^+^ and we will note that these sorts of I-V curves have been seen by several other groups, such as the example from Estacion et al. (2006) (**Figure 2A**). Referring again to the barrier-and-well models of cation permeation by Estacion et al. (2006), these fully recapitulated channel blockade by external Ca^2+^ and the voltage-dependence of Na^+^/Ca^2+^ permeability ratios but it is notable that they did not reproduce the flat regions negative to -60 mV or between 0 and +40 mV (**Figure 2B**). Those authors wrote, “*Specifically, I-V relationships generated by the model do not exhibit the slight ‘hump’ in the current seen around 0 mV or the flattening of inward current seen at potentials more negative than -60 mV…. Overall, however, the I-V relationships generated by the model are remarkably similar to those derived experimentally*.”

**FIGURE 3:**
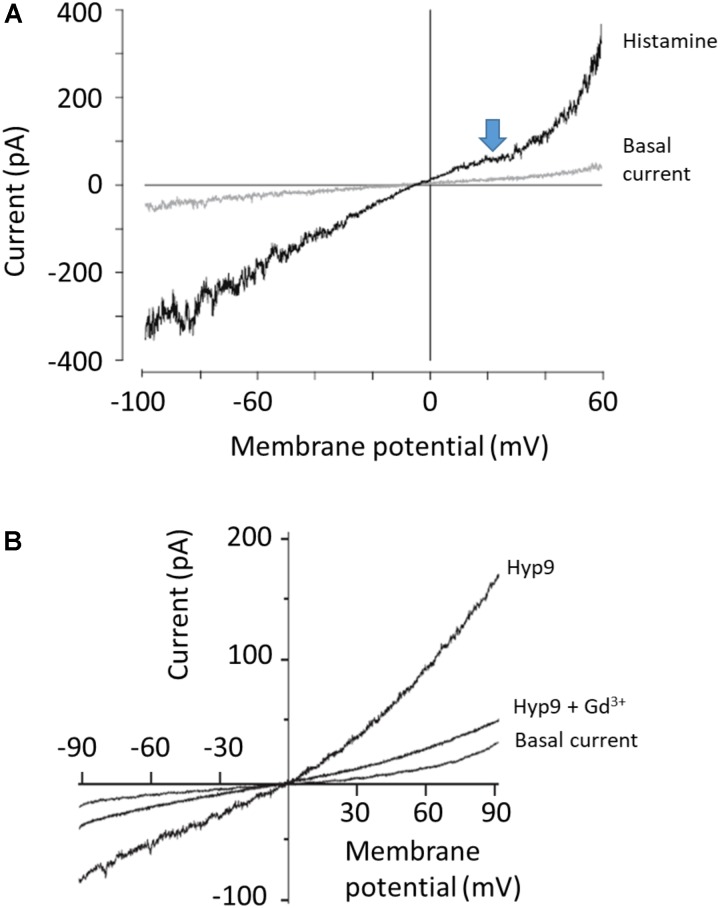
Examples of less complex I-V curves for TRPC6 channels. **(A)** Whole-cell recording of TRPC6 channels expressed in HEK293 cells co-expressing H1 histamine receptors, originally published by Dietrich et al. (2003). Currents are shown before and after application of histamine as indicated. Note flattening of I-V curve between 0 and +40 mV (marked by blue arrow that we have added) but not at negative membrane potentials, where the I-V curve is essentially straight. Bath solutions contained 140 mM Na^+^, 2 mM Ca^2+^, and 1 mM Mg^2+^, and Cs^+^ was the only monovalent cation in the recording pipette solution. **(B)** Perforated patch whole-cell recordings of TRPC6 channels expressed in HEK293 cells, originally published by Leuner et al. (2010). Current shown is before application of any activator (basal current), after application of 10 μM of Hyp9, a phloroglucinol derivative of hyperforin, and in the presence of 10 μM Hyp9 + 100 μM Gd^3+^, as indicated. Bath solution contained 140 mM Na^+^, 2 mM Ca^2+^, and 1 mM Mg^2+^. The only monovalent charge carrier in the recording pipette was Cs^+^. Note that there is not flattening in the I-V curve at any membrane potential. We have re-lettered the axes and traces. Figures are used with permission from the publishers.

While these types of highly complex I-V curves with multiple inflection points have been seen in many papers, it is important to note that this behavior is not recapitulated in every study. For example, **Figure 3A** shows recordings of cloned TRPC6 channels, again expressed in HEK293 cells, from an important and widely cited study (Dietrich et al., 2003). The recordings were made before and after TRPC6 channels were activated by histamine acting on H1 receptors that were co-expressed in the cells. The blue arrow that we have added calls attention to the now familiar flattening of the curve between 0 and +40 mV. However, the rectification at negative membrane potentials (between -60 and -100 mV) is *not* present. The trace shows considerable stochastic fluctuations reflecting channel opening and closing negative to -70 mV (also seen in the studies of Inoue et al., 2001; Estacion et al., 2006) but rectification at the negative membrane potentials is not present. In other words, this recording does not look exactly like the previous two examples. Sometimes I-V curves reported in the literature are even less complex than that, as shown in **Figure 3B**, which again shows recordings from an HEK293 cells transfected with TRPC6 (Leuner et al., 2010). TRPC6 was activated by a phloroglucinol analog of DAG. In this recording, except for slight outward rectification seen through the range of positive membrane potentials there is no inflection point or flattening between 0 and +40 mV or at negative membrane potentials. There are many other examples of traces like that from TRPC6 channels expressed in HEK293 cells (Koitabashi et al., 2010; Shen et al., 2011). In some cases the I-V curves were generated from pulses rather than ramps, but that should not matter.

**FIGURE 4:**
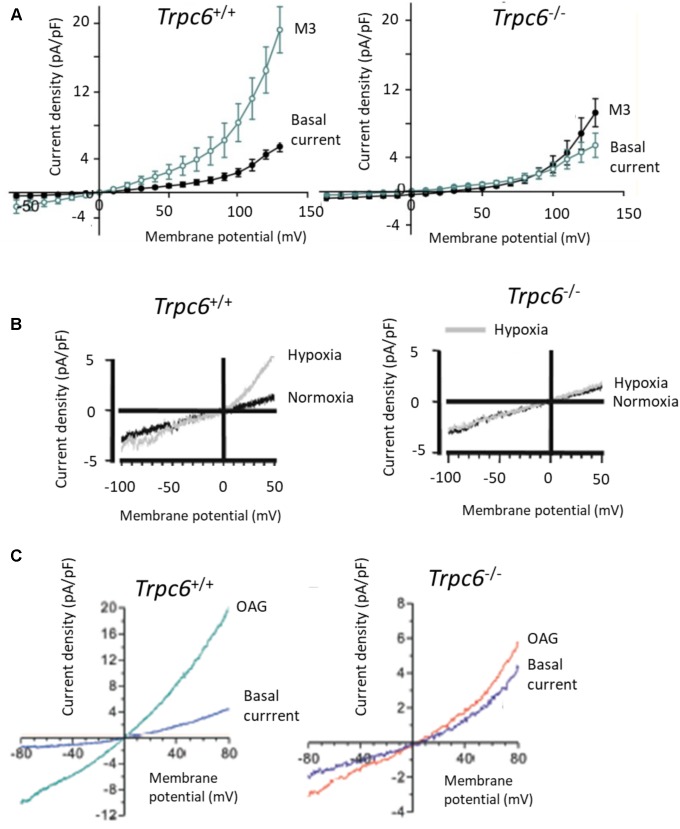
TRPC6 I-V curves in primary cell isolated from Trpc6^+/+^ and Trpc6^-/-^ mice. **(A)** I-V curves from primary alveolar macrophages activated by (*R*)-roscovitine, which stimulates production of DAG in those cells (Riazanski et al., 2015). **(B)** Activation of TRPC6 by hypoxia in pulmonary arterial smooth muscle cells from mice. Note robust activation of current by hypoxia in wild-type but not in Trpc6^-/-^ mice, originally published by Weissmann et al. (2006). **(C)** Activation of currents by 100 μM OAG in primary podocytes, originally published by Eckel et al. (2011). Note that the ordinates are plotted at different scales. In each of these cases there is some outward rectification at quite positive membrane potentials but in no case is there flattening of the I-V curves of the sort shown previously. Used with permission of the publishers.

So far we have only shown examples of recordings of TRPC6 channels in heterologous expression systems. We will next show some examples of recordings from endogenously expressed TRPC6 channels in various cell types. **Figure 4A** shows whole-cell recordings from primary mouse alveolar macrophages, with I-V curves generated from responses to voltage pulses. The currents to the left are from wild-type mice, whereas the ones to the right are from *Trpc6^-^*^/^*^-^* mice. Currents were activated by M3, a derivative of (*R*)-roscovitine that stimulates production of DAG in those cells (Riazanski et al., 2015). There are many other published TRPC6 I-V curves that look like this, especially from primary cells, including many from our laboratory (Anderson et al., 2013; Roshanravan and Dryer, 2014; Roshanravan et al., 2016). For example, **Figure 4B** shows a whole-cell recording from a pulmonary artery smooth muscle cell (PMAC) showing activation of TRPC6 in response to hypoxia (an important observation for pulmonary physiologists) (Weissmann et al., 2006). Note that there is no flattening anywhere in the I-V curve, and that the hypoxia-evoked currents were not seen in *Trpc6^-^*^/^*^-^* mice. It is also worth noting that this recording was from the same group that published the traces in **Figure 3A**. These workers have made many seminal contributions to the literature on TRPC6 and we will show another recording from this group further below. A similar pattern was seen in other recordings of native TRPC6 channels from other smooth muscle preparations (Dietrich et al., 2005). **Figure 4C** shows recordings from primary podocytes cultured from *Trpc6*^+/+^ and *Trpc6^-^*^/^*^-^* mice (Eckel et al., 2011). TRPC6 was activated by OAG, and the OAG activated currents are considerably larger in cells from wild-type animals compared to cells from *Trpc6^-^*^/^*^-^* mice. Again, there is no flattening at any part of the I-V curves.

## Trpc6 Permeation and Gating in Excised Membrane Patches

**FIGURE 5:**
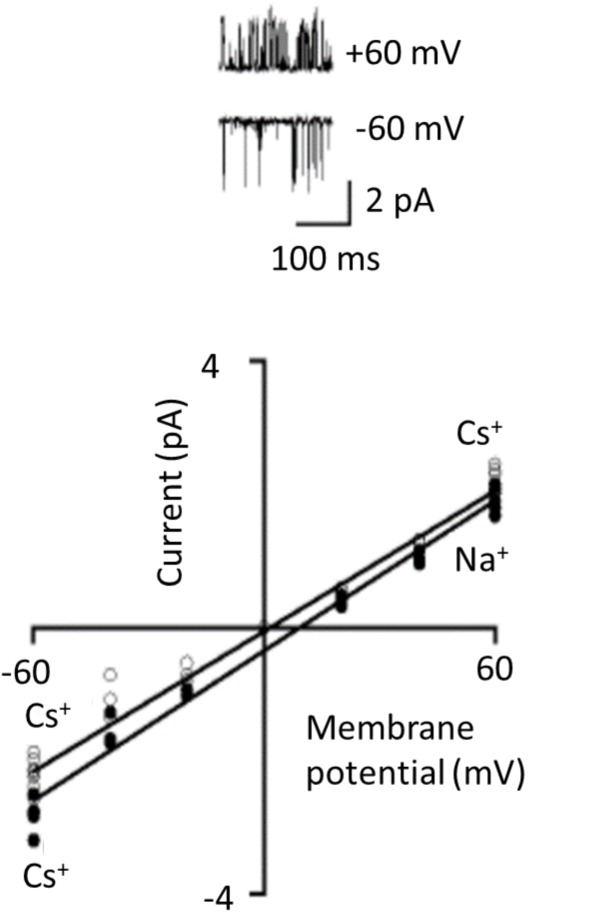
TRPC6 channels observed in excised inside-out patches. These recordings were originally published by Hofmann et al. (1999) and they demonstrated that several essential aspects of transduction to TRPC6 channels are membrane-delimited. TRPC6 was expressed heterologously in CHO-K1 cells along with H1 histamine receptor, and TRPC6 was activated by histamine. The traces are unitary currents through TRPC6 at +60 and –60 mV as indicated. I-V curves generated from these kinds of recordings show no indication at all of rectification, even though the recordings were carried out a range of membrane potentials where one would expect to see it. Linear I-V relationship occurred with either Cs^+^ or Na^+^ as charge carriers in the bath solution. We have re-lettered the axes and traces for clarity. Used with permission of the publishers.

Complex rectification may not even be an intrinsic property of TRPC6 channels. Instead it could arise from extrinsic factors that inhibit the channel in a voltage-dependent manner. In this regard, whether or not robust rectification is seen in TRPC6 seems to depend on the recording configuration used to study the channels. This can be seen in one of the landmark studies on TRPC6 channels, in this cased expressed heterologously in CHO-K1 cells and activated by histamine acting on co-expressed H1 receptors (Hofmann et al., 1999). In whole-cell recordings using ramp voltage commands, marked flattening of the I-V curve was prominent between 0 and +40 mV, strikingly similar to the example shown in **Figure 3A**. Single TRPC6 channels were also detected in excised inside-out patches, which was a crucial observation since it implied that the factors needed to sustain channel activation are membrane delimited (which is not seen for channels in which the physiological activators are cytosolic). However, in excised patches there was no sign of rectification in the TRPC6 I-V curve regardless of whether the bath solution contained 120 mM Cs^+^ (analogous to the conditions typically used in whole-cell recordings) or 120 mM Na^+^ (**Figure 5**). At a series of voltages between -60 and +60 mV the points on the I-V relationship appear follow a straight line in both conditions and that is how the authors plotted the data. From the slopes of the plots they concluded that the channels have unitary conductance of 35 pS (with Na^+^ in the bath) or 37 pS (with Cs^+^ in the bath). While the significance of this result for membrane-delimited transduction to TRPC6 has long been recognized, another implication is that complex rectification may not be an intrinsic property of the channel at all because after the membrane is excised from the cell (thereby removing channels from contact with the cytosol) the rectification behavior is not really discernible. While it is harder to draw firm conclusions on I-V relationships in patches that are not excised, it is worth noting that rectification was not seen in cell-attached patch recordings from podocytes in isolated glomeruli (Ilatovskaya et al., 2014).

## Conclusion

It should now be clear that the nature of the rectification(s) seen in I-V curves is not a reliable criterion for stating whether a current is carried by TRPC6 or not. Rather, it seems to depend (at least in part) on what cell is being studied, and whether the membrane is still in contact with the cytosol. These differences are seen even in recordings from the same investigators made in different systems (Hofmann et al., 1999; Dietrich et al., 2003, 2005; Weissmann et al., 2006). It must be emphasized that the biochemical and biophysical bases for the rectification that undoubtedly can and often does occur is simply not understood. Very slight changes in channel conformation that result in slight displacements of charged residues within the pore could have major effects on the forces and impediments permeant ions are subjected to as they cross the membrane under different driving forces. Such changes could occur as a result of changes in the fluidity of the surrounding bilayer, through interactions with other proteins and auxiliary subunits, or even as a result of soluble molecules in the cytosol. To give one example, endogenous polyamines, such as spermine and spermidine are known to induce rectification in cationic channels that disappears after patch excision (Lopatin et al., 1994; Kim et al., 2016). TRPC6 channels interact with a large number of different proteins (Bouron et al., 2016) and in most cases the functional significance of the interactions are not known but it is not unreasonable to predict that some of these could affect permeation properties.

In addition, there is no particular reason to consider heterologous expression systems, where the most complex I-V curves are most typically seen, as somehow more definitive for defining what the properties of a channel “should” be. This is equally true for studies of permeation, gating, post-translational modification, or trafficking. We have already seen that different groups obtain quite different I-V curves for TRPC6 channels expressed in what are nominally HEK293 cells, and there is no question that gene expression patterns in cell lines drift over time, especially when they have been maintained independently for thousands of passages in different laboratories and/or subjected to stable transfection procedures that required a subsequent selection. Moreover, it has long been known that some HEK293 cell lines express various TRPC subunits endogenously (Garcia and Schilling, 1997; Groschner et al., 1998; Wu et al., 2000), and that non-transfected HEK293 cells are capable of quite complex store- and receptor-operated Ca^2+^ flux pathways (Bugaj et al., 2005). The possibilities for interaction may be greater than is commonly assumed. One sign of this is that gating behavior in TRPC channels can depend on expression level in a heterologous system, suggesting that this can result in changes in the stoichiometry of channel complexes being examined (Vazquez et al., 2003). In this regard, it is worth noting that TRPC3 has a much higher level of constitutive activity than TRPC6 owing to the dual glycosylation of TRPC3 (Dietrich et al., 2003). Therefore TRPC3/TRPC6 heteromers could in principle yield fairly wide range of I-V curves depending on the exact stoichiometry (and their distributions). TRPC1 also forms complexes with TRPC6, resulting in reduced Ca^2+^ permeability of the resulting complex (Storch et al., 2012). In fact, the only way to address these issues with some degree of certainty would be to express TRPC6, TRPC6/TRPC1, and TRPC6/TRPC3 concatemer cDNAs in a heterologous expression system that unambiguously does not express TRPC channels. We are aware of one attempt to do this that was unsuccessful for technical reasons related to amplifying these concatemers in *E. coli* (A. Dietrich, personal communication). Alternatively, one would need to undertake very high resolution co-immunoprecipitation studies in heterologous systems along the lines of those carried out for the TRPC1/TRPC4/TRPC5 family in mouse brain (Bröker-Lai et al., 2017).

Given all this, what properties should be considered definitive for determining if a particular current is carried by channels that require TRPC6 subunits? Normally one would expect the currents to be eliminated or reduced in animals or cell lines in which TRPC6 is eliminated (by genetic ablation) or reduced (by various RNA knock-down methods). In heterologous expression systems, the currents should not be detectable in cells transfected with empty vectors. The currents should be blocked by the appropriate non-selective inhibitors, such as the pan-TRP inhibitor SKF-96365 and by micromolar concentrations of La^3+^. A stronger argument emerges when currents are blocked by more selective inhibitors. Among these, SAR7334 is particularly useful as it blocks TRPC6 at low nanomolar levels (IC_50_ around 10 nM), blocks TRPC3 and TRPC7 at orders of magnitude higher concentrations (IC_50_ around 280 nM), and has no inhibitory effect on any other known channel at concentrations as high as 10 μM (Maier et al., 2015). Other quite specific blockers are emerging (Urban et al., 2016; Häfner et al., 2018). One further expects TRPC6 to be activated by appropriate agonists, such OAG or flufenamic acid (FFA), although it should be noted that OAG is notoriously prone to oxidation and is not easy to use in experiments; and in our hands FFA, while a quite useful compound, is better described as a partial agonist since in whole-cell recordings from podocytes the maximum currents that we observed in response to FFA are always smaller than those evoked by OAG. Of course, DAG analogs also activate TRPC3 (Hofmann et al., 1999). Obviously there can be complications even with these strategies. For example, knocking out TRPC6 can result in compensatory or even over-compensatory up-regulation of TRPC3 on some genetic backgrounds (Dietrich et al., 2005; Kim et al., 2018). Pharmacological approaches must always be undertaken with awareness of the compound specificity or lack thereof. However, a combination of experimental approaches in which genetic, biochemical and pharmacological data converge to indicate that a particular macroscopic current requires TRPC6 subunits should be accepted at face value even if the currents do not show complex rectification. Ultimately agonists, antagonists, and antibodies need to be validated in appropriate knockout models, such as the one originally described by Dietrich et al. (2005).

Up to now we have focused on the permeation and rectification properties of TRPC6 as a means of identifying the channel in a particular cell, because it has occasionally been supposed that TRPC6 channels must by definition show a particular I-V relationship. However, it is also useful to discuss the physiological significance of the I-V and permeation properties of TRPC6. As a general rule, the I-V properties of an ion channel in any given cell are only physiologically relevant at membrane potentials that the cell might actually experience. In other words, if an epithelial cell is never at +40 or -80 mV, then the I-V properties of TRPC6 at those membrane potentials simply don’t matter for that cell (but might be significant for a different cell type). In this context we can make a distinction between excitable cells (especially neurons and striated muscle cells) and non-excitable cells (such as epithelia or immune cells, but also smooth muscle undergoing certain modes of contraction). Consider, for example, a cardiac myocyte, which during diastole may be sitting at -90 mV. If there is substantial rectification at that membrane potential (as in the example in **Figure 1**), then current through TRPC6 at that phase of the cardiac cycle would be reduced compared to what would be seen if the channel showed the properties in **Figure 2**. This would tend to reduce depolarizing drive that might exist on top of that produced by other channels. In sinoatrial node cells, where TRPC6 is expressed robustly along with TRPC3 and TRPC1 (Ju et al., 2007), rectification at negative membrane potentials (if it were present) would tend to reduce its contribution to pacemaker depolarization compared to what would occur if no rectification was present. Similarly, rectification between 0 and +40 mV, which is a physiological range, would tend to result in reduced Ca^2+^ influx or other cation flux during peak and plateau phases of action potentials. This would tend to reduce Ca^2+^ influx during that phase compared to what would occur if no rectification was present. In this regard, the I-V properties of TRPC6 channels in sinoatrial node cells have not been established. Smooth muscle cells exhibit incredibly diverse electrical behavior and a host of Ca^2+^ signaling mechanisms (Berridge, 2008). Some exhibit spontaneous pacemaker activity, and most are capable of discharging regenerative action potentials where the presence or absence of rectification would affect resulting cation influx through TRPC6. However contraction in smooth muscle can also occur with very small changes in membrane potential, as in the rabbit ear artery (Droogmans et al., 1977), and in those cases the presence or absence of rectification of TRPC6 between 0 and +40 mV would not affect cation flux. Most non-excitable cells, such as, e.g., epithelia or immune cells, operate over a much narrower range of membrane potentials, and one would certainly not expect that any TRPC6 rectification at positive membrane potentials (or lack thereof) would have physiological significance for those cells. On the other hand, in non-excitable cells, TRPC6 and related channels may represent a major source of Ca^2+^ influx themselves (as opposed to simply providing depolarization for activation of voltage-sensitive Ca*_v_* channels). In that cases there may be mechanisms in place to prevent excessive depolarization that would compromise Ca^2+^ permeability of the channels (Estacion et al., 2006). In other words, the rectification properties of TRPC6 may be tuned to its function in a particular cell type. Further, we do not know if these rectification properties can be modulated by other extrinsic factors and therefore change within a cell over time. As yet these topics almost completely unexplored.

## Author Contributions

SD and EK conceived the work together. SD wrote the text and prepared the figures.

## Conflict of Interest Statement

The authors declare that the research was conducted in the absence of any commercial or financial relationships that could be construed as a potential conflict of interest.
